# On the Meaning of Affinity Limits in B-Cell Epitope Prediction for Antipeptide Antibody-Mediated Immunity

**DOI:** 10.1155/2012/346765

**Published:** 2012-11-14

**Authors:** Salvador Eugenio C. Caoili

**Affiliations:** Department of Biochemistry and Molecular Biology, College of Medicine, University of the Philippines Manila, Room 101, Medical Annex Building, 547 Pedro Gil Street, Ermita, Manila 1000, Philippines

## Abstract

B-cell epitope prediction aims to aid the design of peptide-based immunogens (e.g., vaccines) for eliciting antipeptide antibodies that protect against disease, but such antibodies fail to confer protection and even promote disease if they bind with low affinity. Hence, the Immune Epitope Database (IEDB) was searched to obtain published thermodynamic and kinetic data on binding interactions of antipeptide antibodies. The data suggest that the affinity of the antibodies for their immunizing peptides appears to be limited in a manner consistent with previously proposed kinetic constraints on affinity maturation in vivo and that cross-reaction of the antibodies with proteins tends to occur with lower affinity than the corresponding reaction of the antibodies with their immunizing peptides. These observations better inform B-cell epitope prediction to avoid overestimating the affinity for both active and passive immunization; whereas active immunization is subject to limitations of affinity maturation in vivo and of the capacity to accumulate endogenous antibodies, passive immunization may transcend such limitations, possibly with the aid of artificial affinity-selection processes and of protein engineering. Additionally, protein disorder warrants further investigation as a possible supplementary criterion for B-cell epitope prediction, where such disorder obviates thermodynamically unfavorable protein structural adjustments in cross-reactions between antipeptide antibodies and proteins.

## 1. Introduction

 Antibody-mediated immunity is the basis of most conventional approaches to immunization, which protect against or treat disease by means of antibodies that are either endogenous (i.e., produced via active immunization, notably through the administration of vaccines that elicit antibody responses) or exogenous (i.e., acquired via passive immunization through the administration of preformed antibodies from some external source, such as a human or animal donor). Historically, these approaches have been developed and pursued mainly for the prevention and control of communicable infectious diseases viewed as public-health problems, which is ever more crucial to adequately address current and anticipated global-health challenges posed by emerging and reemerging pathogens that cause pandemics and panzootics (both of which may be inextricably linked in cases of zoonoses such as avian and swine influenza) [1]. Yet, the envisioned practical applications of antibody-mediated immunity increasingly include therapy for and prophylaxis against diseases such as cancer and hypertension that have traditionally been regarded as lifestyle related rather than infectious [2, 3] although some of these diseases may be at least partly due to infectious agents (e.g., oncogenic viruses) that are thus important targets of antibody-mediated immunity. In a very general sense, possible targets of antibody-mediated immunity include virtually all biomolecules regardless of origin and are often dichotomously categorized as being either self (i.e., autologous, or host associated) or nonself (e.g., pathogen associated), but the distinction is potentially misleading in that a typical vertebrate host normally becomes colonized by microbes acquired from its environment early in life to form a complex biological system (i.e., an ecosystem-like superorganism) comprising both the host and its symbiotically associated microbes [4], such that the concept of self arguably encompasses the host and microbial components of the system.

Antibody-mediated immunity targets a biomolecule as an antigen (i.e., substance recognized by the immune system) through a molecular-recognition process whereby a paratope (i.e., antigen-binding site on an antibody) binds an epitope (i.e., submolecular structural feature actually recognized on the antigen). In this context, the epitope is recognized as a B-cell epitope (rather than a T-cell epitope, for which the overall recognition process is much more elaborate and involves a T-cell receptor instead of antibody) [5]. Accordingly, B-cell epitope prediction is the computational identification of putative B-cell epitopes on antigen structures [6]; in practice, this is usually performed for peptidic (i.e., protein or peptide) antigens on the basis of structural information ranging from amino-acid sequences (as deduced from nucleic-acid sequences) to atomic coordinates (obtained experimentally or in turn from computational analyses of amino-acid sequences) [7]. From the perspective of generating protective antibody-mediated immunity while also avoiding adverse antibody-mediated reactions, B-cell epitope prediction is potentially useful if it correctly anticipates biological effects of paratope-epitope binding interactions, so as to guide the pursuit of beneficial rather than harmful clinical outcomes. Ideally, this would enable the design of safe and efficacious vaccines, which presupposes the ability to accurately model the in vivo kinetics of both antibody buildup and affinity maturation (i.e., the microevolutionary process by which antibody affinity can be increased through somatic hypermutation among competing B-cell clones in the course of an antibody response) insofar as clinical outcomes (e.g., protection against or enhancement of infection) reflect the interplay of antibody concentration and antibody affinity. A more computationally tractable task is the design of immunogens (e.g., peptide-based constructs) to produce antibodies or derivatives thereof (e.g., Fab fragments) that protect against disease via passive immunization, which circumvents the complexities and limitations of endogenous antibody production. Antibodies may bind antigens and thereby exert biological effects, which may occur directly due to binding per se (e.g., via direct neutralization of biological activity, as in the inhibition of enzymes or the blocking of pathogen adhesion molecules) or indirectly due to the activation of downstream immune effector mechanisms such as complement pathways and opsonization-facilitated phagocytosis [8]. These mechanisms are typically protective, but they may paradoxically promote pathogenesis under certain circumstances.

Biological outcomes of immunization are contingent upon thermodynamic and kinetic constraints on antibody-antigen interactions, as exemplified by context-dependent roles of antibodies in mediating either protection against or enhancement of infection. The latter phenomenon has been observed among infections due to a wide variety of pathogens including taxonomically diverse viruses [9, 10], notably enveloped viruses such as HIV [11, 12] and flaviviruses (e.g., dengue and West Nile viruses [13, 14]), and even bacteria and protozoa [15, 16]. Among enveloped viruses, this often occurs when virions are incompletely coated by IgG-class antibodies, which favors enhanced infection by promoting viral adsorption onto host cells via capture of virion-bound IgG by Fc-*γ* receptors while still permitting fusion between viral and cellular membranes [17]. HIV infection of monocytes has thus been mathematically modeled [17], thereby recapitulating the empirical observation that the enhancement of infection is favored at low antibody concentrations and by low-affinity antibody binding; hence, even high-affinity antibody binding may enhance infection below a certain threshold antibody concentration that increases as affinity decreases.

Protective antibody-mediated immunity is favored over antibody-mediated enhancement of infection by increasing either or both antibody concentration and affinity, yet this is practically feasible only up to certain limits. Even below the solubility limit of antibodies in aqueous solution, buildup of supraphysiologic antibody concentrations in vivo may produce hyperviscosity syndrome [18]. Moreover, high antibody concentrations may be difficult to attain via active immunization although this limitation might be overcome by passive immunization (e.g., with purified monoclonal antibodies). The practically feasible maximum antibody concentration, as dictated either by safety considerations or by actual outcomes of immunization, thus defines a minimum affinity below which protective antibody-mediated immunity is an unrealistic prospect. At the same time, affinity itself is subject to physicochemical and physiological constraints that limit its magnitude [19, 20]. These considerations motivate the present work, which aims to clarify their implications for B-cell epitope prediction as applied to the generation of antipeptide antibodies that protect against disease.

## 2. Theory and Methods

### 2.1. Upper Bounds for Affinity

 The affinity of antibodies for antigens is often quantitatively expressed as the association constant *K*_*A*_ (i.e., affinity constant) or equivalent dissociation constant *K*_*D*_, such that

(1) $$\begin{array}{c} K_{A}=\frac{1}{K_{D}}\\ K_{A}=\exp\left(-\frac{\Delta G}{RT}\right), \end{array}$$

where Δ*G* is the free energy change of association, *R* the gas constant, and *T* the temperature. As Δ*G* is ultimately a function of biomolecular structure, *K*_*A*_ may, in principle, be estimated from structural information. Where only antigen structure is known, this may be partitioned into B-cell epitopes for which Δ*G* may be approximated from their solvent-accessible surface area (ASA) within the framework of structural energetics [21, 22] under certain simplifying assumptions (e.g., that the epitope is completely buried upon binding by the paratope, which loses approximately the same amounts of apolar and polar ASA as the epitope in the process [23, 24]). The value of Δ*G* thus obtained corresponds to a theoretical upper bound for affinity where the structural complementarity between epitope and paratope approaches that between close-packed internal surfaces of a natively folded protein. However, this may greatly exceed the affinity realized during B-cell development [19].

Naive B cells express surface immunoglobulins for receptor-mediated endocytosis of antigens as an initial step towards recruiting T-cell help for activation, which in turn prompts B-cell proliferation with mutation of immunoglobulin-coding genes to diversify the paratopic repertoire. This entails competition among B cells for T-cell help, with B-cell survival favored by rapid endocytosis of antigens for presentation to helper T cells. The endocytic antigen-uptake rate may be increased either by increasing the on-rate constant *k*_*on*_ for antigen capture or by decreasing the off-rate constant *k*_*off*_ for antigen escape. As both rate constants are related by

(2) $$\begin{array}{c} K_{A}=\frac{k_{\mathrm{on}}}{k_{\mathrm{off}}}, \end{array}$$

mutations that increase the antigen-uptake rate also increase the affinity for antigen according to (1), for which reason the mutation phase of B-cell development is known as affinity maturation. Hence, increases in affinity for antigen tend to favor B-cell clonal selection, but only up to a certain ceiling level as may be explained in terms of limits on both *k*_*on*_ and *k*_*off*_ during affinity maturation [19], considering that the upper bound for *K*_*A*_ is defined by the upper bound for *k*_*on*_ and the lower bound for *k*_*off*_ according to (2).

For binding of interaction partners *A* and *B*, the upper bound for *k*_*on*_ is the on-rate constant for diffusion-limited collisional encounters, as given by

(3) $$\begin{array}{c} k_{\mathrm{on}}^{\max}=4\pi a\left(D_{A}+D_{B}\right)\left(\frac{N}{1000}\right), \end{array}$$

where *a* is the encounter distance, *D*_*A*_ and *D*_*B*_ are the diffusion constants, and *N* is Avogadro's number (i.e., 6.02 × 10^23^ mol^−1^). Using (3), *k*_*on*_^max^ is obtained in M^−1^ s^−1^ for *a* in cm and for *D*_*A*_ and *D*_*B*_ in cm^2^ s^−1^ [25]. For binding of small protein antigens by antibodies in solution, *k*_*on*_^max^ is estimated to be in the range of 10^5^ to 10^6^ M^−1^ s^−1^ [26, 27], and antibodies in general are thus unlikely to have much higher values of *k*_*on*_^max^ [19]. For capture of IgG-class antibodies from solution by immobilized antigens in surface plasmon resonance (SPR) studies, where the antigen diffusion constant is practically zero, *k*_*on*_^max^ may be estimated from (3) using an encounter distance of 1.57 × 10^−8^ cm and an antibody diffusion constant of 4 × 10^−7^ cm^2^ s^−1^, yielding a value of 4.75 × 10^7^ M^−1^ s^−1^ [25].

To estimate the lower bound for *k*_*off*_ during affinity maturation, endocytic antigen uptake may be modeled to a first approximation with classical Michaelis-Menten kinetics applied to transmembrane transport, in which case the Michaelis-Menten constant is given by

(4) $$\begin{array}{c} K_{M}=\frac{\left(k_{\mathrm{off}}+k_{\text{in}}\right)}{k_{\mathrm{on}}}, \end{array}$$

where *k*_in_ is the rate constant for endocytic internalization of surface immunoglobulin-bound antigen. As *K*_*M*_ is numerically equivalent to the antigen concentration at which the steady-state rate of antigen internalization is half-maximal, a decrease in *K*_*M*_ confers a competitive advantage upon B cells to the extent that they are thus enabled to internalize antigen more rapidly than other B cells. Consequently, *k*_*on*_ may approach *k*_*on*_^max^ (from (3)) in the course of affinity maturation. However, *k*_*off*_ is unlikely to decrease much further below *k*_in_ as the gain in competitive advantage would then be negligible [19]; according to (4), *K*_*M*_ approaches the lower limit of *k*_in_/*k*_*on*_ for values of *k*_*off*_ much lower than *k*_in_, in which case the values of *K*_*M*_ are approximately uniform such that none is distinctly advantageous over the others. Considering the reported half-life of 8.5 min for surface immunoglobulins prior to their endocytosis on Epstein Barr virus-transformed B-lymphoblastoid cells [28], the lower bound for *k*_*off*_ during affinity maturation is estimated to be in the range of 10^−4^ to 10^−3^ s^−1^ under the assumption that two to three surface-immunoglobulin half-lives is the upper limit beyond which increased immune-complex stability confers no competitive advantage [19].

Competition among B cells for endocytic uptake of antigens is thus a plausible mechanism that limits the emergence of antibodies with low *k*_*off*_ during affinity maturation. A related mechanism has been proposed that may likewise limit the emergence of antibodies with low *k*_*off*_, namely, sequestration of antigens by antibodies in highly stable immune complexes that limits the availability of antigens for endocytic uptake by B cells [29]. Notwithstanding the operation of these mechanisms, the theoretical upper bound for affinity might still be closely approached where optimal complementarity between epitopes and paratopes arises fortuitously (e.g., by initial rearrangement of germline immunoglobulin-gene sequences) prior to any affinity maturation [19], and artificial selection processes (e.g., with yeast display) may transcend the limits of in vivo affinity maturation [20].

Apart from the upper bound for affinity per se and the kinetic constraints imposed during affinity maturation, an additional consideration arises in relation to cross-reaction of antipeptide antibodies with protein antigens. Typically, this involves a peptide whose sequence forms part of a cognate protein; as an immunogen, the peptide may elicit antipeptide antibodies, but these may cross-react with the protein with very low affinity. Such problems are the concern of B-cell epitope prediction for generating antipeptide antibodies that exert biological effects by cross-reacting with proteins. A major challenge therein is the difficulty of predicting the affinity with which antipeptide antibodies cross-react with proteins. If such cross-reaction is to result in biological effects, it must occur with sufficiently high affinity with the proteins in biologically relevant molecular contexts (e.g., in native conformational and oligomerization states, possibly as integral components of supramolecular complexes such as biological membranes). Relevant experimental results reported thus far have mostly been limited to qualitative assessment of the binding per se without biological correlates [30]; yet these data nonetheless suggest that cross-reaction of antipeptide antibodies with proteins tends to occur with lower affinity than the corresponding reaction of the antibodies with the immunizing peptides. This would be consistent with thermodynamically unfavorable structural adjustments (e.g., unfolding of proteins to conformationally mimic their peptide counterparts) during cross-reactions; and if this is actually the case for antipeptide antibodies, their affinity in reactions with immunizing peptides represents a plausible practical upper bound for their affinity in cross-reactions with proteins.

With regard to antipeptide antibodies that cross-react with protein antigens, three upper bounds for affinity may thus be discerned: the first pertains to binding per se in the reaction of antipeptide antibodies with their immunizing peptides, the second, to binding realized during affinity maturation, and the third, to cross-reaction of the antipeptide antibodies with protein antigens. Among these three, the first is necessarily an upper bound for the second (as the first is never exceeded during affinity maturation) but not for the third (as cross-reaction with the protein may be thermodynamically more favorable than reaction with the immunizing peptide, e.g., due to lower conformational entropy of the protein relative to the peptide); however, the third is unlikely to exceed the second where cross-reaction entails thermodynamically unfavorable structural adjustment (e.g., protein unfolding to conformationally mimic the immunizing peptide). Hence, physicochemical constraints on both affinity maturation and cross-reaction are expected to limit the affinity of antipeptide antibodies for protein antigens and, consequently, the capacity of such antibodies to mediate protective immunity (e.g., to protect against rather than enhance infection). Knowledge of such constraints is therefore potentially useful for B-cell epitope prediction in order to avoid overestimating the affinity of cross-reaction. 

### 2.2. Retrieval and Processing of Epitope Data

**Figure 1:**
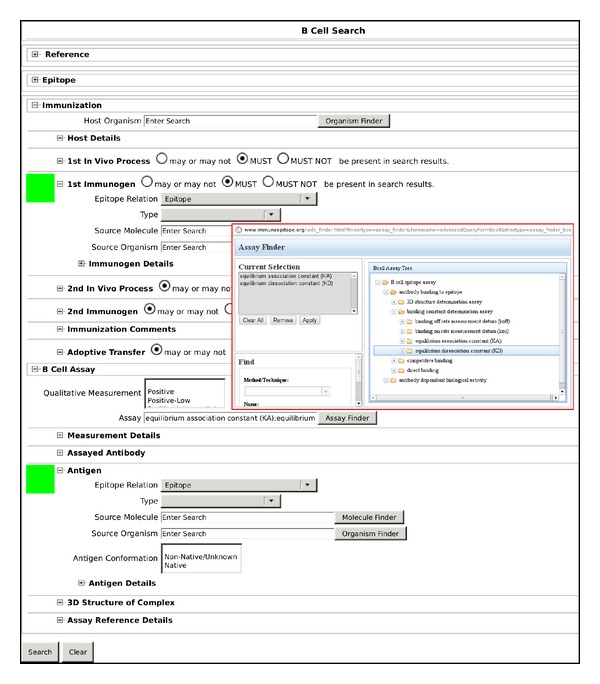
IEDB B Cell Search facility interface (http://www.immuneepitope.org/advancedQueryBcell.php). Example shown corresponds to primary search for thermodynamic data (see main text for full explanation). Green squares along left margin mark user options selected from pull-down menus, for restricting searches by data fields of the type “Epitope Relation;” upper and lower green squares, respectively, mark options for “1st Immunogen Epitope Relation” (set to “Epitope” for both primary and secondary searches”) and “Antigen Epitope Relation” (set to either “Epitope” for primary searches or “Source antigen” for secondary searches). Inset with red border contains screenshot of Assay Finder pop-up window (activated by clicking the Assay Finder button, located along bottom edge of inset), which facilitates the selection of search-appropriate assay-type categories using the B-cell Assay Tree (shown in right panel of inset).

 To further investigate the limits on affinity of antipeptide antibodies for immunizing peptides and for cognate protein antigens, published thermodynamic and kinetic data were retrieved on binding interactions of antipeptide antibodies, using the Immune Epitope Database and Analysis Resource (IEDB; http://www.immuneepitope.org/) [31]. Relevant curated data were retrieved from IEDB by means of searches conducted with its B Cell Search facility (Figure 1), which returns records that each pertain to a B-cell assay for a particular epitope. Each record thus returned contains multiple data fields, several of which are defined in relation to the concepts of “1st Immunogen” (i.e., immunogen administered to produce antibodies) and “Antigen” (i.e., antigen used in the B-cell assay).

Searches were restricted by the data fields named “1st Immunogen Epitope Relation” and “Antigen Epitope Relation” (hereafter referred to as the immunogen and antigen fields, resp.). For both thermodynamic and kinetic data, primary and secondary searches were conducted, which respectively retrieved data on reactions of antipeptide antibodies with peptides and on cross-reactions of the same antibodies with proteins. The primary searches retrieved records for which the epitope comprised both immunogen and antigen, such that both immunogen and antigen fields had the value “Epitope”. The secondary searches retrieved records for which the epitope also comprised the immunogen but formed only a part of the antigen, such that the immunogen field had the value “Epitope” while the antigen field had the value “Source antigen”. Additionally, each search was further restricted to return only those records containing either thermodynamic or kinetic data by filtering with respect to B-cell assay type (represented by the data field named “Assay”). Such filtering was performed using the Assay Finder feature of the B Cell Search facility.

Within the Assay Finder pop-up window, the B-cell assay tree was navigated to view the available assay-type categories under the subheading of “binding constant determination assay” (itself under the subheading of “antibody binding to epitope”), and appropriate selections of the said assay-type categories were defined for filtering in order to retrieve only those records matching one of the selected assay types. For thermodynamic data, the selected assay-type categories were “equilibrium association constant (KA)” and “equilibrium dissociation constant (KD)”; each of these categories comprised assay types of calorimetry, enzyme-linked immunosorbent assay (ELISA), fluorescence immunoassay (FIA), radioimmunoassay (RIA), and surface plasmon resonance (SPR), all of which were further qualified as having measurements expressed in units of either “[1/nM]” (for “KA”) or “[nM]” (for “KD”). For kinetic data, the selected assay-type categories were “binding on rate measurement datum (kon)” and “binding off rate measurement datum (koff)”; each of these categories comprised assay types of FIA and SPR, both of which were further qualified as having measurements expressed in units of either “[M^−1^ s^−1^]” (for “*k*_*on*_”) or “[s^−1^]” (for “*k*_*off*_”).

A total of four searches (i.e., a primary and a secondary search each for thermodynamic and kinetic data) were conducted between 16 and 18 July 2012, and the search results were downloaded as IEDB full-format comma-separated value (CSV) files comprising B-cell epitope records. Subsequent processing of records focused mainly on the data field named “Quantitative measurement” whose numeric value was a thermodynamic or kinetic measurement. Records were excluded from further consideration in cases wherein the data field named “Measurement Inequality” contained an inequality symbol (either “<” or “>”, indicating that the numeric value was a lower or upper bound rather than a point estimate) or for which the epitope was nonpeptidic (i.e., wherein the data field named “Epitope Object Type” had a value of “Non-peptidic” instead of “Linear peptide”).

Records retrieved through each primary search were processed before those of the corresponding secondary search in order to facilitate pairing of counterpart records that essentially differed from one another only in the antigen field (whose value was “Epitope” for the primary search and “Source antigen” for the secondary search); records retrieved through a secondary search were processed only where they were thus found to be counterparts of retained records from the corresponding primary search. For kinetic data, records were retained only where data were available on both the on- and off-rate constants for a particular binding interaction. For each record that was ultimately retained, the numeric value was compared with that originally reported in the underlying literature reference; where discrepancies were found, the values from literature were used for subsequent analysis, and the discrepancies were reported to the maintainers of IEDB.

Records containing thermodynamic data were segregated by units of measurement into two categories, each comprising data on either association constants or dissociation constants in units of 1/nM or nM, respectively. Corresponding association constants were calculated from dissociation constants according to (1), and all association constants were expressed in units of 1/M. Records on both association and dissociation constants were ranked in order of decreasing affinity. The ranked records were inspected for equal or nearly equal association-constant values, for which the underlying records and literature references were reviewed to explore the possibility of data redundancy; where a pair of such values was found to represent equivalent association and dissociation constants, the record for the dissociation constant was deemed redundant and was thus excluded from further analysis. The underlying literature references were also reviewed to confirm that all data included in the final analysis described antibody-antigen binding interactions themselves rather than conditions (e.g., concentrations of chaotropic agents) under which the interactions were studied.

Records containing kinetic data were segregated by units of measurement into two categories, comprising data on either on- or off-rate constants in units of M^−1^ s^−1^ or s^−1^, respectively. Records pertaining to on- and off-rate constants from a common literature reference were reviewed in conjunction with the literature reference to identify pairs of corresponding on- and off-rate constants pertaining to the same binding interaction. For each pair of rate constants thus identified, the records on thermodynamic data were searched for a corresponding record on an association constant (or equivalent dissociation constant) also pertaining to the same binding interaction and related to the rate constants according to (2).

## 3. Results and Discussion

### 3.1. Affinity

**Figure 2:**
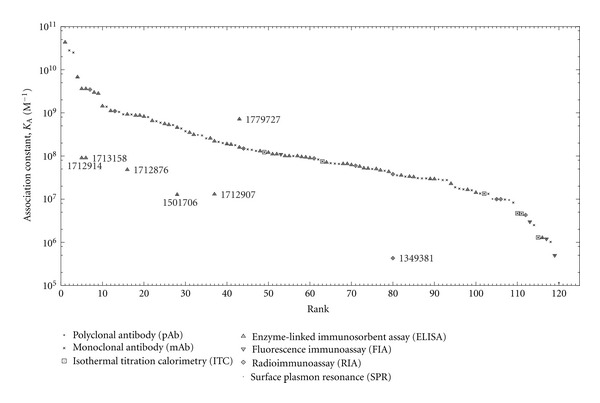
Affinities of antipeptide antibodies for their epitopes. Each data point is plotted as a pair of superposed symbols for antibody type (polyclonal or monoclonal) and B-cell assay type (as indicated in the legend). Unlabeled data points are affinity ranked and represent reactions of antipeptide antibodies with peptides (Tables 1 and 2). Other data points are labeled by IEDB B-Cell ID number and represent cross-reactions of antipeptide antibodies with proteins. Data points sharing the same abscissa value pertain to the same antibody and other B-cell assay conditions except the antigen.

For reactions of antipeptide antibodies with peptides, a dataset of 120 records on affinities of antipeptide antibodies for their peptidic epitopes was assembled (Figure 2), comprising 56 records on polyclonal antibodies and 64 records on monoclonal antibodies. (Two records, with IEDB B-Cell IDs 1603957 and 1603959 and both containing quantitative measurements with IEDB assay type units of “KD [nM],” were excluded from the dataset because their data pertained to concentrations of the chaotropic agent ammonium thiocyanate required to dissociate 50% of bound antibody from immobilized peptide antigen in an ELISA [32], as a measure of avidity rather than an actual dissociation constant.) Reference data on these records are presented in Tables 1 and 2 for association constants above and below the median value, respectively. The lowest and highest association constants were 1.15 × 10^5^ and 4.30 × 10^10^ M^−1^, respectively, with a median of 8.57 × 10^7^ M^−1^. The highest association constant was thus lower than the ceiling value of 4.75 × 10^11^ M^−1^ expected for affinity maturation, as calculated using (2) from values of 4.75 × 10^7^ M^−1^ s^−1^ for *k*_*on*_ [25] and 10^−4^ s^−1^ for *k*_*off*_ [19] (noting that the *k*_*on*_ value thus cited is appropriate for solid-phase immunoassays wherein immobilized antigens capture IgG-class antibodies from solution, which is the case for most data in Figure 2 including the highest association constant). These data are compatible with an affinity ceiling during affinity maturation in vivo as previously suggested on kinetic grounds [19]. However, only the monoclonal-antibody data correspond to homogeneous antibody-molecule populations; the polyclonal-antibody data represent averages for heterogeneous antibody-molecule populations, each of which may thus exhibit variation in affinity for antigen among its constituent antibody molecules such that a subset thereof might actually exceed the proposed affinity ceiling. Furthermore, although the artificial-selection processes of monoclonal-antibody production are deliberately biased towards obtaining high-affinity clones, this fails to guarantee that the highest-affinity clones are indeed ultimately isolated (e.g., because hybridoma survival may be poorly correlated with affinity), which cautions against assuming that the monoclonal-antibody data provide stronger support than the polyclonal-antibody data for the proposed affinity limit, especially in view of the presently observed overlap between monoclonal and polyclonal antibodies in their affinity-value ranges.

For cross-reactions of antipeptide antibodies with proteins, seven additional records were found on affinities of antipeptide antibodies for protein source antigens containing the epitope sequences of the immunizing peptides, such that each additional record had a counterpart pertaining to the same antibody in the dataset for reactions of antipeptide antibodies with peptides (Figure 2). Association constants were typically more than an order of magnitude lower for cross-reactions with proteins than for the corresponding reactions with peptides, except in the case of a monoclonal antibody (rank 43 in Figure 2 and Table 1) whose association constant was actually higher for cross-reaction with protein than for reaction with peptide. This monoclonal antibody was produced by immunization with an epitope consisting of two cross-linked peptides corresponding to residues 395–402 and 402–411 (cross-linked at Gln 398 and Lys 406) of the C-terminal region on human fibrin *γ*-chain [53], in which case lower conformational entropy of the epitope as part of the cognate protein rather than the immunizing peptide may at least partly account for higher affinity of cross-reaction with protein relative to reaction with immunizing peptide. Overall, these results are consistent with a trend towards thermodynamically unfavorable structural adjustments upon cross-reaction with protein that lead to lower binding affinity relative to reaction with immunizing peptides, but the exceptional case of the human fibrin epitope demonstrates the possibility of higher affinity with cross-reaction.

### 3.2. Kinetics

**Figure 3:**
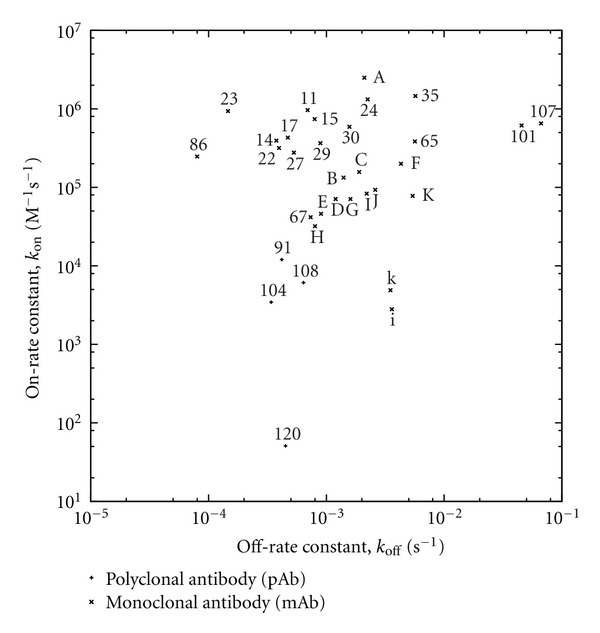
On- and off-rates of antipeptide antibodies binding their epitopes, obtained by surface plasmon resonance (SPR). For reactions of antipeptide antibodies with peptides, data points are labeled either by affinity rank in Figure 2 for corresponding IEDB records on affinity data or, where such records were not found, alphabetically with uppercase letters in order of decreasing affinity [76]. For cross-reactions of antipeptide antibodies with protein [76], data points are labeled with lower-case letters (i and k) matching the uppercase letter labels (I and K) of data points for the corresponding reactions of the antibodies with peptide.

 For reactions of antipeptide antibodies with peptides, a dataset of 31 rate-constant record pairs containing data on corresponding on- and off-rate constants from surface plasmon resonance (SPR) studies was assembled (Figure 3; Table 3), comprising four record pairs on polyclonal antibodies and 27 record pairs on monoclonal antibodies. On the basis of underlying literature references and (2), corresponding records on affinity data (Figure 2; Tables 1 and 2) were found for most of the rate-constant record pairs, except in the cases of 11 record pairs on monoclonal antibodies (Figure 3, labels A through K); where the affinity data were published, they had been computed directly from their corresponding rate constants according to (2) rather than obtained directly (i.e., by another independent experimental means). The lowest and highest on-rate constants were 5.1 × 10^1^ and 2.49 × 10^6^ M^−1^ s^−1^, respectively, with the latter below the upper bound of 4.75 × 10^7^ M^−1^ s^−1^ for diffusion-limited reaction as calculated in Section 3.1. The lowest and highest off-rate constants were 8.00 × 10^−5^ and 6.65 × 10^−2^ s^−1^, respectively. Most of the data were on immobilized antigens capturing IgG-class antibodies from solution, except for the data points labeled 86 (with the lowest off-rate) and 120 (with the lowest on-rate) in Figure 3, in which cases the data were on immobilized antibodies capturing antigens from solution. If these exceptions are excluded from consideration, the lowest on- and off-rate constants are 3.44 × 10^3^ M^−1^ s^−1^ and 1.46 × 10^−4^ s^−1^, respectively (for the data points labeled 104 and 23 in Figure 3). These data are compatible with a lower bound of 1 × 10^−4^ s^−1^ for off-rate during affinity maturation in vivo as previously suggested [19].

For cross-reactions of antipeptide antibodies with proteins, two additional rate-constant record pairs were found on antipeptide antibodies cross-reacting with a protein source antigen (tobacco mosaic virus protein) containing the peptidic epitope (source antigen residues 110–135; IEDB Epitope ID 94786) of the antibodies [76], such that each additional record had a counterpart pertaining to the same antibody in the dataset for reactions of antipeptide antibodies with peptides (Figure 3). On-rate constants were more than an order of magnitude lower for cross-reactions with protein than for the corresponding reactions with peptide; off-rate constants were either higher or lower for cross-reactions with protein than for the corresponding reactions with peptide. The lower on-rate constants for cross-reaction are consistent with thermodynamically unfavorable structural adjustment to attain complementarity between epitope and paratope.

### 3.3. Data Representativeness and Redundancy

 Despite the attempt to exhaustively retrieve relevant data from IEDB, the datasets thus assembled herein are small, with this problem being worse for the kinetic data. The problem is further compounded by interrelated issues of data representativeness and redundancy. The paucity of data points immediately suggests that the datasets are of limited representativeness in the sense of capturing various combinations of experimental conditions, especially in view of the myriad variables (immunogen structure, immunized species, immunization conditions, cognate antigen structure, assay conditions, etc.) likely to be correlated with immunologic outcomes. Moreover, redundancy is apparent on inspecting for similarities among the IEDB records, each of which represents a B-cell assay that may be unique only with respect to a single variable. For instance, the entire subset of kinetic data labeled with uppercase letters in Figure 3 and Table 3 is on a panel of monoclonal antibodies elicited by a single peptide and assayed for binding the same peptide (having a 26-mer sequence derived from tobacco mosaic virus protein [76]), such that each underlying B-cell assay is unique only with respect to its particular monoclonal antibody. Here, data redundancy might be approached by reducing all the data for the entire panel to some representative (e.g., average) value for each rate constant (i.e., placing the entire panel on par with a single polyclonal-antibody data point), but this would entail loss of information (e.g., obscuring the observation that data point A corresponds to the highest on-rate constant). Furthermore, each member of a monoclonal-antibody panel (and for that matter each distinct idiotype of a polyclonal antibody sample) might bind a unique site on a peptide that has been operationally defined as a single B-cell epitope according to IEDB curation guidelines for lack of data on antigenic fine structure (in the sense of high-resolution epitope mapping) [77]; even if the unique sites overlapped to some extent, each could itself still be regarded as a B-cell epitope [5]. This underscores the difficulty of accounting for redundancy in B-cell epitope datasets. Simply reasoning by analogy, for example, to the management of redundancy in general-purpose protein-structure datasets [78–80], data might be inappropriately conflated for B-cell assay records sharing identical or otherwise similar peptide sequences, thus ignoring the possibility of yet unresolved antigenic fine structure and of radically divergent antigenic properties arising from seemingly minor sequence differences (e.g., even in a single chemical group [81]).

Undoubtedly, the problems of data representativeness and redundancy in B-cell epitope datasets must be rigorously formulated and resolved accordingly to facilitate further development of B-cell epitope prediction tools, but such a task is well beyond the scope of the present study. If at all the datasets herein are somehow representative of antibody-antigen interactions in general, this may be by virtue of thermodynamic and kinetic constraints (e.g., during affinity maturation) that immunization processes are typically subject to, which nonetheless calls for further validation on the basis of more numerous and diverse prospectively acquired experimental data as these become available.

### 3.4. Implications

 Considering the thermodynamic and kinetic data included in the present work, two key observations emerge. First, affinity of antipeptide antibodies for proteins is likely to be overestimated if computed as a theoretical upper bound for binding per se without regard for affinity maturation. Second, affinity of antipeptide antibodies for proteins tends to be lower than for the immunizing peptides used to elicit the antibodies. These observations serve to clarify crucial problems encountered in B-cell epitope prediction that seeks to quantitatively estimate affinity of antipeptide antibodies for proteins. One problem thus clarified is the difficulty of estimating the maximum affinity of antipeptide antibodies for immunizing peptides which is realized during immunization; although this maximum affinity may be estimated from antigen structure by means of structural energetics [23, 24], the highest affinity that is actually realized may be much lower due to kinetic constraints on affinity maturation [19, 20] and also to suboptimal immunization conditions such as choice of adjuvant [41, 43, 44, 57, 59, 64, 69, 75, 82]. A related problem is the difficulty of estimating affinity of the antipeptide antibodies for proteins in view of the structural differences between the immunizing peptides and the proteins [30, 83]; even if the affinity of the antibodies for the immunizing peptides is known, it may differ markedly from the affinity for cognate proteins of the peptides, which may be much lower due to thermodynamically unfavorable structural adjustments of cross-reaction.

The abovementioned problems could be addressed in several ways. In particular, affinity maturation could be accounted for in B-cell epitope prediction by an appropriate ceiling on predicted affinity values. Furthermore, immunization conditions (e.g., adjuvants) could be optimized so as to maximize the affinity of elicited antipeptide antibodies. In certain cases, however, the ceiling on predicted affinity values may be lower than previously suggested on the basis of endocytic uptake of univalent antigen [19], particularly for multivalent antigens that can cross-link surface immunoglobulins on B cells. Immunoglobulin cross-linking by multivalent antigens entails multiple simultaneous epitope-paratope binding interactions, in which case high avidity (i.e., overall strength of binding) may result even where the individual epitope-paratope binding interactions are each of low affinity. Surface-immunoglobulin cross-linking may thus enable efficient endocytic uptake of multivalent antigens by B cells even in the setting of low-affinity epitope-paratope interactions, and it may also favor B-cell activation more directly via transmembrane signal-transduction pathways [84, 85]. In view of this added complexity posed by multivalent antigens, which include immunogens that comprise typical peptide-carrier protein conjugates and multiple antigenic peptides, the outcome of higher affinity might be favored by avoiding surface-immunoglobulin cross-linking during affinity maturation (e.g., by immunizing with a construct containing only one copy of the B-cell epitope that is the intended target of the antibody response). More generally, limitations of natural affinity maturation in vivo might be overcome by artificial selection methods (e.g., based on yeast display [20]) or by protein engineering of paratopes for improved complementarity to target epitopes. As to the problem of predicting affinities of cross-reactions between antipeptide antibodies and their envisioned protein targets, this might be at least partly addressed by basing predictions on similarities between each immunizing peptide and its corresponding region on the protein target, with emphasis on conformation and on overall physical accessibility to antibodies. This approach may be readily feasible in cases where the immunizing peptide and its corresponding protein region share the same sequence and are intrinsically disordered (i.e., unfolded and behaving as dynamic random coils with rapidly fluctuating backbone conformations [86]) while the protein region is located on an antibody-accessible site (e.g., exposed on the surface of an extracellular protein domain), such that the antipeptide antibodies may bind the protein with essentially the same affinity as for the peptide insofar as thermodynamically unfavorable structural adjustments would be unnecessary for the protein to mimic the peptide. Although the classical concept of completely folded native protein structures identifies dynamic disorder with denatured states, intrinsic protein disorder has more recently been observed in native states of an increasingly diverse repertoire of proteins among all domains of life, with the extent of disorder ranging from short protein segments to full-length proteins [86]. An antibody-accessible natively disordered protein region may thus be structurally mimicked by a similarly disordered peptide of identical sequence, and if the peptide bears a B-cell epitope that is bound by a complementary paratope with sufficient affinity, the peptide may elicit antipeptide antibodies that bind the peptide and the protein region with similar affinities via a process of paratope-induced epitope folding whereby the epitope becomes immobilized in a conformation that is readily adopted in both the peptide and the cognate protein. Existing B-cell epitope prediction methods may actually account for this possibility to some extent (e.g., using flexibility parameters, or implicitly via machine learning). Thus utilizing information on dynamic disorder broadens the scope of B-cell epitope prediction based on structural similarity between peptides and their cognate proteins, as exemplified by prior work on identifying *β*-turns as markers of epitope structure [87] considering that they may be present in both peptide and protein structure [88] particularly where they form early in the course of the folding process [89].

The preceding considerations are applicable to B-cell epitope prediction for generating antipeptide antibodies that exert biological effects by cross-reacting with proteins, both for active immunization (e.g., with peptide-based vaccines) and for passive immunization (e.g., with antipeptide antibodies from exogenous sources). For each candidate protein target of antipeptide antibodies, the target structure (i.e., the target protein as it occurs in its biologically relevant conformational state and higher-order structural context [30]) may be partitioned into candidate B-cell epitopes for which antibody affinity could be estimated [23, 24], either with or without the assumption of a ceiling on affinity during affinity maturation [19, 20]. This affinity-ceiling assumption would be made only where affinity maturation would actually be relevant to the envisioned practical application (e.g., active immunization with peptide-based vaccines, but not passive immunization with monoclonal antipeptide antibodies), and the exact value of the affinity ceiling would depend on factors such as host characteristics (especially those pertaining to B-cell development) and details of the immunization process (including adjuvants and the nature of the immunogen, e.g., univalent versus multivalent). To evaluate each candidate B-cell epitope for potential utility, an affinity cutoff value could be established for cross-reaction of antipeptide antibodies with the epitope as part of the target structure, such that the epitope would be deemed potentially useful only if the estimated antibody affinity were to exceed the cutoff value. The cutoff value itself might be determined in relation to some estimated maximum antibody concentration (e.g., based on projected postvaccination outcomes) necessary to achieve a certain biological outcome (e.g., protection against rather than enhancement of viral infection, as mathematically modeled for enveloped viruses [17]). If a sufficient number of potentially useful candidate epitopes is thus found even with an affinity-ceiling assumption for affinity maturation in vivo, the epitopes could be incorporated into a peptide-based vaccine for active immunization; otherwise, the affinity cutoff value could be adjusted downwards (e.g., by raising the maximum antibody concentration to a physically realistic yet reasonably safe level), and potentially useful epitopes that might then be found could be incorporated into a peptide-based immunogen for generating antibodies to mediate passive immunization (e.g., by the administration of antipeptide monoclonal antibodies). In cases where the affinity-ceiling assumption were to preclude the identification of suitable candidate epitopes, this assumption could be dropped with the proviso that artificial affinity selection (e.g., based on yeast display) or antibody engineering would enable realization of the predicted affinities. Additionally, protein disorder might yet serve as a supplementary predictive criterion (e.g., by focusing exclusively on candidate epitopes that are predicted to be intrinsically disordered in the target structure), so as to avoid uncertainties of modeling thermodynamically unfavorable structural adjustment among the target proteins as they mimic the immunizing peptides. Bearing in mind this theoretical consideration, protein disorder warrants further investigation on the basis of additional data as these become available.

The practical significance of affinity limits in B-cell epitope prediction is thus clearly evident in relation to the problem of antibody-mediated enhancement of infection. At a host-population level, mass immunization (e.g., by natural infection, vaccination, or passive acquisition of antibodies) may initially confer protective antibody-mediated immunity to infection by attaining sufficiently high antibody concentrations among many hosts, but subsequent shifts from protective to infection-enhancing effects may occur as antibody concentrations decrease over time. In light of the preceding considerations, B-cell epitope prediction is meaningful if it quantitatively captures pertinent antibody-mediated biological effects in a context-dependent manner that informs clinical and public-health decisions, possibly by demonstrating the inadequacy of antibody-based approaches in particular situations (e.g., where antibody affinity falls below some critical threshold for practical utility).

**Figure 4:**
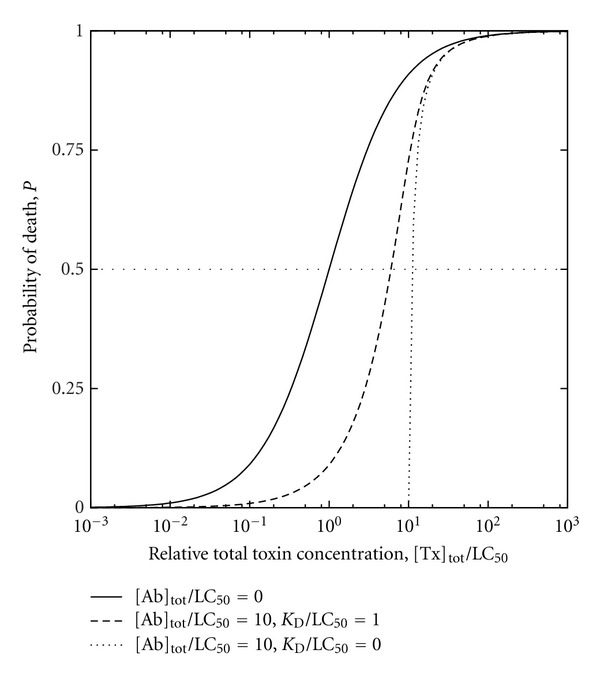
Representative theoretical dose-response curves for toxin of median lethal concentration LC_50_ and antitoxin antibody of dissociation constant *K*_*D*_, based on (5) through (8). Toxin and antibody concentrations are expressed in terms of respective total values [Tx]_tot_ and [Ab]_tot_ comprising free and bound species in binding equilibrium.

**Figure 5:**
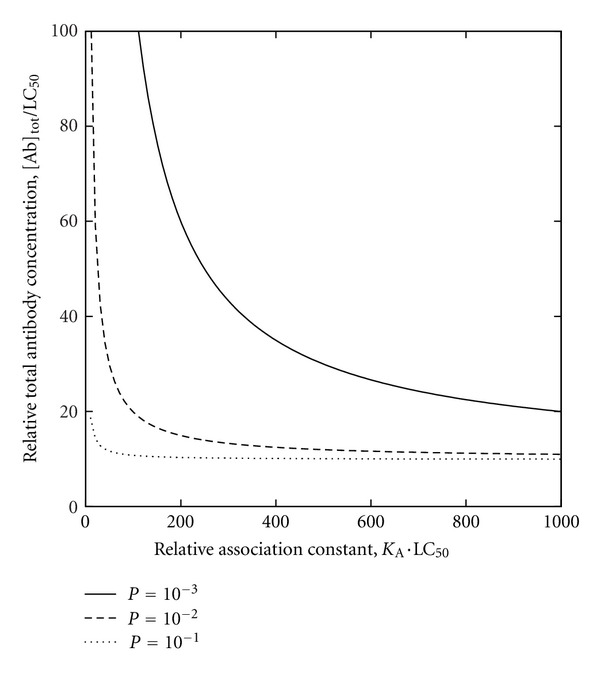
Contour map based on dose-response relationships of Figure 4 for [Tx]_tot_/LC_50_ = 10 (i.e., total toxin concentration tenfold greater than LC_50_), depicting *P* as a function of both antibody affinity (expressed in terms of the association constant *K*_*A*_; cf. (1)) and antibody concentration (i.e., minimum required antibody concentration of Figure 6).

**Figure 6:**
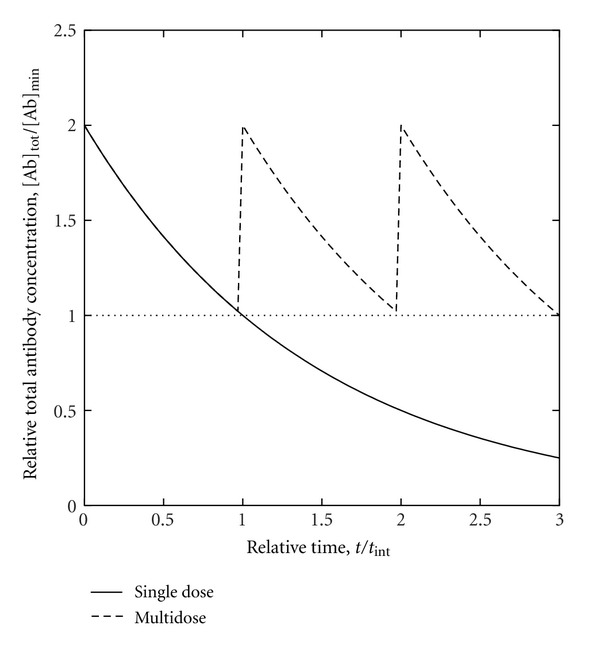
Passively-acquired antibody concentration as a function of time. After administering a dose of antibody to raise the total antibody concentration [Ab]_tot_ above the minimum required antibody concentration [Ab]_min_, [Ab]_tot_ eventually falls below [Ab]_min_ unless more antibody is administered within the maximum dosing interval *t*_int_.

More generally, biological effects of antibody-mediated immunity can be analyzed in relation to both antibody affinity and antibody concentration in order to appreciate the practical implications of B-cell epitope prediction. To clarify this approach, an instructive example is that of a nonreplicating toxin bound by an antibody, such that binding of the toxin by the antibody neutralizes the toxin while both the affinity and the concentration of the antibody in vivo (e.g., in plasma) are independent variables. Toxin biological activity can be expressed within a toxicologic dose-response framework as the killed fraction of a host population following the administration of a standardized toxin dose (possibly normalized per unit body mass) to each member of the population, for a given affinity-concentration pair (i.e., combination of antibody-affinity and antibody-concentration values, both held to be uniform over the entire population). For each affinity-concentration pair, a dose-response curve can be constructed by plotting the killed fraction (as the ordinate) against the toxin dose (as the abscissa). Granted that each dose-response curve is a strictly monotonically increasing function of typical sigmoidal form extending from the origin (i.e., zero killed fraction at zero toxin dose) and having a unique point at 50% (i.e., half-maximal) killed fraction, the toxin dose corresponding to the latter point is the median lethal dose LD_50_ for the particular affinity-concentration pair. The LD_50_ may be expressed as the median lethal concentration LC_50_ (e.g., in a body fluid or in-vitro culture medium), which facilitates analysis in relation to antibody concentration. Without loss of generality, this can be illustrated using a simple model featuring rapid-equilibrium reversible binding of toxin by antibody, toxicity due only to free (i.e., unbound) toxin, and a sigmoidal dose-response curve in the absence of antibody, such that the curve is shifted towards increased survival by either or both increased antibody concentration and increased antibody affinity for toxin. The toxin-antibody dissociation constant *K*_*D*_ (cf. (1)) may thus be written in terms of the concentrations of toxin Tx, antibody Ab, and toxin-antibody complex TxAb, either as

(5) $$\begin{array}{c} K_{D}=\frac{\left[\text{Tx}\right]\left[\text{Ab}\right]}{\left[\text{TxAb}\right]} \end{array}$$

or equivalently as

(6) $$\begin{array}{c} K_{D}=\frac{\left({\left[\text{Tx}\right]}_{\text{tot}}-\left[\text{TxAb}\right]\right)\left({\left[\text{Ab}\right]}_{\text{tot}}-\left[\text{TxAb}\right]\right)}{\left[\text{TxAb}\right]}, \end{array}$$

where each symbol with enclosing square brackets ([]) denotes the molar concentration of the corresponding species and the subscript (tot) denotes the total for free and bound forms of a species. Likewise, the probability *P* of toxin-induced death may be written either as

(7) $$\begin{array}{c} P=\frac{1}{1+\text{L}{\text{C}}_{50}/\left[\text{Tx}\right]} \end{array}$$

or equivalently as:

(8) $$\begin{array}{c} P=\frac{1}{1+\text{L}{\text{C}}_{50}/\left({\left[\text{Tx}\right]}_{\text{tot}}-\left[\text{TxAb}\right]\right)} \end{array}$$

such that the dose-response relationship for toxin lethality may thus be represented by plotting *P* against total toxin concentration expressed relative to LC_50_ (Figure 4). Increasing either or both affinity and concentration consequently increases the LC_50_ (as more toxin is required to kill half the population). The protective benefit attributed to a particular affinity-concentration pair can be quantitatively expressed relative to zero antibody concentration (e.g., as the difference between the LC_50_ with and without antibody), and a plot of concentration against affinity can be constructed for affinity-concentration pairs that confer equal protective benefit (Figure 5). From a biomedical perspective, critical points on the plot would include those corresponding to physical and physiologic upper bounds on affinity and concentration; the physical upper bounds are the theoretical maximum affinity for paratope-epitope binding and the solubility limit of antibody in plasma while the physiologic upper bounds are the expected maximum affinity realized through affinity maturation and the normal endogenous-antibody concentration. Between the normal endogenous-antibody concentration and the solubility limit of antibody in plasma, additional thresholds can be defined (e.g., for pathologic conditions due to plasma hyperviscosity resulting from excessively high antibody concentrations). If B-cell epitope prediction is performed to estimate antibody affinities for putative neutralization epitopes of the toxin [23], the estimated affinities can in turn be used to calculate the antibody concentrations required to achieve predefined levels of protective benefit (i.e., increase in LD_50_ relative to zero antibody concentration), and the concentrations can be assessed in terms of feasibility (from a purely technical standpoint) and acceptability (with attention to health risks, costs, and other nontechnical considerations). Where continuous long-term protection might be sought, the assessment would entail the calculation of dosing intervals for the administration of either exogenous antibody for passive immunization (e.g., as schematically depicted in Figure 6) or booster doses of vaccine for active immunization. If active immunization were thus deemed unrealistic or impractical as a means to attain adequate affinity or concentration, passive immunization might be considered as an alternative (possibly with artificial selection methods that circumvent the physiologic affinity limit); if even passive immunization were deemed unrealistic or impractical, yet other alternatives (e.g., pharmacologic) might be explored. 

Similar analyses can be conducted for more complicated cases, notably communicable infectious diseases (in which case ID_50_, the median infectious dose of a pathogen, can replace or supplement LD_50_ as a parameter of interest). For these diseases, a key epidemiologic consideration is the emergent property of herd immunity (i.e., overall resistance of a host population to the spread of an infectious disease, even where a fraction of hosts lacks protective immunity as individuals), which allows for some degree of fault tolerance (e.g., for incomplete population coverage by immunization programs and for variability in the protection afforded by individual host immune responses). In cases where antibody-dependent enhancement of infection occurs, the prospect of realizing benefit must be weighed against the risk of causing harm; depending on exactly how this is accomplished, the possibility of harm may argue against antibody-mediated immunity attained through active rather than passive immunization (considering that the effects of active immunization are much more difficult to reverse) or even against antibody-mediated immunity altogether (considering that entirely cell-mediated immunity may be a viable alternative in certain instances, as suggested by the observation that hosts unable to mount antibody responses can nonetheless successfully resist viral infection by means of T-cell responses [90]). In the last case, B-cell epitope prediction might thus serve to identify putative epitopes that ought to be excluded from, rather than included in, immunogens designed as vaccines. This may be especially relevant where the rational design of vaccine immunogens to elicit protective antibodies is of questionable feasibility, as exemplified by the open problem of HIV vaccine design [91].

In all such analyses, the casting of antibody-mediated immunity in terms of benefit, harm, risk, cost, and allied concepts inevitably introduces a normative dimension into the discussion of B-cell epitope prediction, the meaning of which is then understood as contingent upon interrelated issues of ethics, economics, and society at large. Hence, antibody affinity for binding putative epitopes ultimately enters into moral calculations under forms of aggregative consequentialism such as utilitarianism (which seeks to maximize aggregate utility in the sense of overall wellbeing) and prioritarianism (which is similar to utilitarianism but employs weighting schemes to prioritize those who are relatively worse-off in terms of individual wellbeing). This is conditioned by the application of ethical principles such as nonmaleficence (i.e., avoidance of causing harm), which derives from the medical precept of *primum non nocere* (first do no harm) and is conceptually related to the precautionary principle (i.e., assigning the burden of proof, in the interest of sustainability, to proponents of activities that may threaten health and environment) [92]. Comprehension of these issues is necessary to rationally approach major global-health challenges such as the efficient implementation of vaccination programs, especially with regard to timely allocation of limited vaccine supplies [93, 94].

## 4. Conclusions

Affinity of antipeptide antibodies for their immunizing peptides appears to be limited in a manner consistent with kinetic constraints on affinity maturation, and cross-reaction of these antibodies with proteins tends to occur with even lower affinity. These observations serve to better inform B-cell epitope prediction for generating antipeptide antibodies that cross-react with proteins, particularly to avoid overestimation of affinity for both active and passive immunization. Whereas active immunization is subject to limitations of affinity maturation in vivo and of the capacity to accumulate endogenous antibodies, passive immunization may transcend such limitations, possibly via artificial affinity-selection processes and protein engineering. In addition to affinity, protein disorder warrants further investigation as a possible supplementary criterion for B-cell epitope prediction where such disorder obviates thermodynamically unfavorable structural adjustments in cross-reactions of antipeptide antibodies with proteins. These considerations could guide the further development of B-cell epitope prediction that is meaningful in relation to biomedical applications insofar as it addresses the biological impact of antibody-mediated immunity in ways that facilitate quantitative evaluation of both benefit and harm, from clinical and public-health perspectives; this is conceivably feasible if based on accurate estimation of antibody affinities for putative epitopes that in turn enables calculation of antibody concentrations required for various biological effects of antibody-mediated immunity, thereby supporting informed decisions to adopt particular strategies (e.g., induction versus avoidance of antibody-mediated immunity, and active versus passive immunization) in the context of a comprehensive theoretical framework that encompasses interrelated technical, ethical, economic, and societal concerns.

## Figures and Tables

**Table 1:** IEDB affinity data, above median.

Rank	B cell ID	Epitope ID	*K*_A_(M^−1^)	Ref. number
1	1713243	123283	4.30 × 10^10^	[33]
2	1694121	119943	2.80 × 10^10^	[34]
3	1377940	54911	2.50 × 10^10^	[35]
4	1502913	22303	6.72 × 10^9^	[36]
5	1712919	123282	3.60 × 10^9^	[37]
6	1713155	123282	3.60 × 10^9^	[33]
7	1662870	111856	3.47 × 10^9^	[38]
8	1710417	123058	2.96 × 10^9^	[37]
9	1710420	123221	2.80 × 10^9^	[37]
10	1772326	131658	1.43 × 10^9^	[39]
11	1313016	4701	1.39 × 10^9^	[40]
12	1349361	11821	1.11 × 10^9^	[41]
13	1636267	104452	1.10 × 10^9^	[42]
14	1313012	4701	1.05 × 10^9^	[40]
15	1313005	4701	9.26 × 10^8^	[40]
16	1712874	125323	9.20 × 10^8^	[37]
17	1313015	4701	9.17 × 10^8^	[40]
18	1349366	7766	8.70 × 10^8^	[41]
19	1244111	18084	8.70 × 10^8^	[43]
20	1349364	11821	8.20 × 10^8^	[41]
21	1313011	4701	8.00 × 10^8^	[40]
22	1487415	7766	6.54 × 10^8^	[44]
23	1789780	105769	6.41 × 10^8^	[45]
24	1278023	54666	5.92 × 10^8^	[46]
25	1244119	15938	5.56 × 10^8^	[43]
26	1349365	7766	5.29 × 10^8^	[41]
27	1313010	4701	5.24 × 10^8^	[40]
28	1502914	22303	4.57 × 10^8^	[36]
29	1803541	7493	4.35 × 10^8^	[47]
30	1313014	4701	3.76 × 10^8^	[40]
31	1487413	11821	3.48 × 10^8^	[44]
32	1487416	7766	3.12 × 10^8^	[44]
33	16285	59318	3.08 × 10^8^	[48]
34	1587464	36959	3.00 × 10^8^	[49]
35	1278024	54666	2.56 × 10^8^	[46]
36	1487414	11821	2.56 × 10^8^	[44]
37	1712912	124998	2.20 × 10^8^	[37]
38	1329743	58132	2.13 × 10^8^	[50]
39	1329922	58132	2.00 × 10^8^	[50]
40	1705153	120407	1.90 × 10^8^	[51]
41	1313385	33796	1.85 × 10^8^	[52]
42	1329915	31002	1.79 × 10^8^	[50]
43	1779729	134133	1.59 × 10^8^	[53]
44	1930562	164463	1.49 × 10^8^	[54]
45	1329916	31002	1.43 × 10^8^	[50]
46	1329731	31002	1.39 × 10^8^	[50]
47	1329918	31002	1.32 × 10^8^	[50]
48	1710412	123058	1.30 × 10^8^	[55]
49	1335178	75791	1.23 × 10^8^	[56]
50	1244124	66382	1.20 × 10^8^	[43]
51	1865651	70070	1.10 × 10^8^	[57]
52	1865652	63967	1.10 × 10^8^	[57]
53	1483242	64541	1.10 × 10^8^	[58]
54	1865649	15938	1.00 × 10^8^	[57]
55	1883894	63967	1.00 × 10^8^	[57]
56	1329744	45673	1.00 × 10^8^	[50]
57	1479672	27725	1.00 × 10^8^	[59]
58	1865628	18084	9.52 × 10^7^	[57]
59	1244126	8267	9.36 × 10^7^	[43]
60	1244234	28937	8.99 × 10^7^	[43]

**Table 2:** IEDB affinity data, below median.

Rank	B cell ID	Epitope ID	*K*_A_(M^−1^)	Ref. number
61	1681389	113966	8.80 × 10^7^	[60]
62	1329745	56122	8.33 × 10^7^	[50]
63	1338803	8067	7.50 × 10^7^	[61]
64	1883893	70070	7.09 × 10^7^	[57]
65	1811868	7491	6.83 × 10^7^	[62]
66	1329928	56122	6.67 × 10^7^	[50]
67	1649813	107849	6.58 × 10^7^	[63]
68	1883884	18084	6.58 × 10^7^	[57]
69	1883887	15938	6.58 × 10^7^	[57]
70	1336690	14958	6.21 × 10^7^	[64]
71	1664695	113966	5.97 × 10^7^	[38]
72	1883888	15938	5.71 × 10^7^	[57]
73	1883895	63967	5.21 × 10^7^	[57]
74	1883892	70070	5.13 × 10^7^	[57]
75	1329924	58132	5.00 × 10^7^	[50]
76	1652921	108291	5.00 × 10^7^	[65]
77	1865650	66382	4.67 × 10^7^	[57]
78	1329930	267	4.55 × 10^7^	[50]
79	1883886	18084	4.31 × 10^7^	[57]
80	1349380	49305	3.80 × 10^7^	[66]
81	1329747	266	3.57 × 10^7^	[50]
82	1883890	66382	3.55 × 10^7^	[57]
83	1329927	56122	3.33 × 10^7^	[50]
84	1772218	131654	3.33 × 10^7^	[39]
85	1336692	19093	3.27 × 10^7^	[64]
86	1697081	103097	3.08 × 10^7^	[67]
87	1329923	58132	3.03 × 10^7^	[50]
88	1329933	266	3.03 × 10^7^	[50]
89	1784467	134492	2.94 × 10^7^	[68]
90	1883891	66382	2.92 × 10^7^	[57]
91	1464180	571	2.88 × 10^7^	[69]
92	1329746	267	2.78 × 10^7^	[50]
93	1329936	45673	2.78 × 10^7^	[50]
94	1336693	3290	2.28 × 10^7^	[64]
95	1329934	266	1.89 × 10^7^	[50]
96	1329932	267	1.75 × 10^7^	[50]
97	1329935	266	1.69 × 10^7^	[50]
98	1336694	19097	1.65 × 10^7^	[64]
99	1329929	56122	1.61 × 10^7^	[50]
100	1652922	108482	1.41 × 10^7^	[65]
101	1922426	162870	1.35 × 10^7^	[70]
102	1335279	75789	1.34 × 10^7^	[56]
103	1329937	45673	1.33 × 10^7^	[50]
104	1464193	30063	1.01 × 10^7^	[69]
105	1636270	104452	1.00 × 10^7^	[42]
106	1484082	7005	9.95 × 10^6^	[71]
107	1922425	162870	9.80 × 10^6^	[70]
108	1464140	30093	9.58 × 10^6^	[69]
109	1329938	45673	8.33 × 10^6^	[50]
110	1335193	74035	4.70 × 10^6^	[56]
111	1335182	75791	4.60 × 10^6^	[56]
112	1459804	6078	4.30 × 10^6^	[72]
113	1245815	68401	3.00 × 10^6^	[73]
114	1352617	24591	2.50 × 10^6^	[74]
115	1335197	74036	1.30 × 10^6^	[56]
116	1705155	120403	1.27 × 10^6^	[51]
117	1245814	68401	1.20 × 10^6^	[73]
118	1352618	24591	1.02 × 10^6^	[74]
119	1245813	68401	5.00 × 10^5^	[73]
120	21742	4467	1.15 × 10^5^	[75]

**Table 3:** IEDB rate-constant data.

Label in Figure 3	B cell ID for on-rate constant	B cell ID for off-rate constant
11	1312987	1313002
14	1312984	1312997
15	1312948	1312990
17	1312986	1313001
21	1312983	1312996
23	1789783	1789790
24	1278965	1278971
27	1312982	1312993
29	1803539	1803540
30	1312985	1313000
35	1278972	1278973
65	1811862	1811867
67	1650289	1650291
86	1697079	1697080
91	1464226	1464233
101	1922428	1922430
104	1464248	1464255
107	1922427	1922429
108	1464204	1464211
120	22186	22187
A	1581476	1581498
B	1581474	1581496
C	1581475	1581497
D	1581473	1581494
E	1581467	1581488
F	1581471	1581492
G	1581473	1581495
H	1581472	1581493
I	1581469	1581490
J	1581470	1581491
K	1581468	1581489
i	1581939	1581944
k	1581940	1581945
